# Pylephlebitis: A Rare Complication of Acute Appendicitis

**DOI:** 10.7759/cureus.31377

**Published:** 2022-11-11

**Authors:** Christine Kim, Vanessa Torres, Olivier Urayeneza, Gudata Hinika

**Affiliations:** 1 Surgery, California Hospital Medical Center, Los Angeles, USA

**Keywords:** portal vein thrombosis, appendicitis, pylephlebitis, intra-abdominal infection, superior mesenteric vein thrombosis

## Abstract

Pylephlebitis is defined as an infective suppurative thrombosis of the portal vein and its tributaries - a rare complication of intra-abdominal infections. It is most commonly seen in patients with diverticulitis and appendicitis. Prompt diagnosis with abdominal ultrasound and computerized tomography (CT) scan along with early and aggressive treatment with broad-spectrum antibiotics is crucial because of its high mortality rates. However, this diagnosis is often missed due to the nature of its nonspecific clinical symptoms. We discuss a case of a 22-year-old male who presented with pylephlebitis as a complication of acute gangrenous appendicitis. The patient was treated successfully with appropriate surgical intervention, antibiotics, and anticoagulation.

## Introduction

Pylephlebitis is defined as an infective suppurative thrombosis of the portal vein and its tributaries [1]. It is a rare complication of intra-abdominal infections, commonly seen in patients with diverticulitis and appendicitis. This condition is associated with a high mortality and morbidity rate mainly due to its nonspecific presentation leading to a delayed or even missed diagnosis. As a result, many cases are diagnosed postmortem [2]. For these reasons, it is highly important to consider it as a differential for any patient presenting with abdominal sepsis. Currently, there are only a handful of reported cases of pylephlebitis with even fewer number of cases following laparoscopic appendectomy. 

In this case, the patient presented with pylephlebitis a week after laparoscopic appendectomy and having completed appropriate antibiotic therapy. Once diagnosed with a new onset complication of pylephlebitis, the patient was successfully treated with appropriate broad-spectrum intravenous (IV) antibiotics and anticoagulation therapy.

## Case presentation

**Figure 1:**
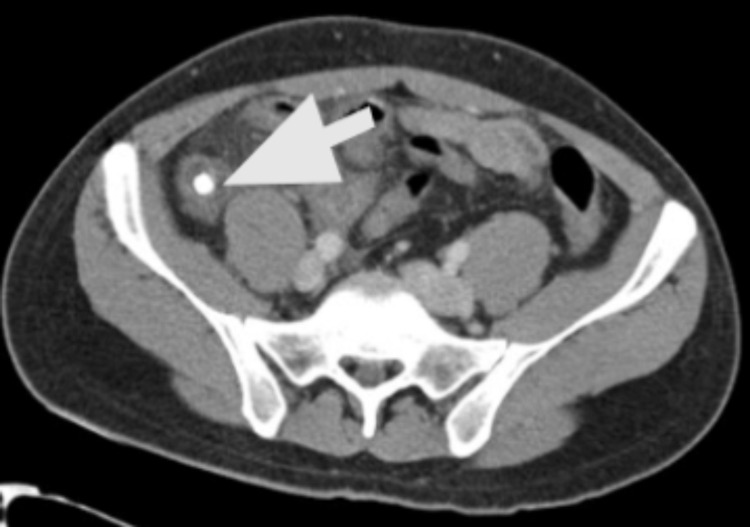
Contrast enhanced CT of the abdomen and pelvis showing 10 mm appendicolith at the base of the appendix

**Figure 2:**
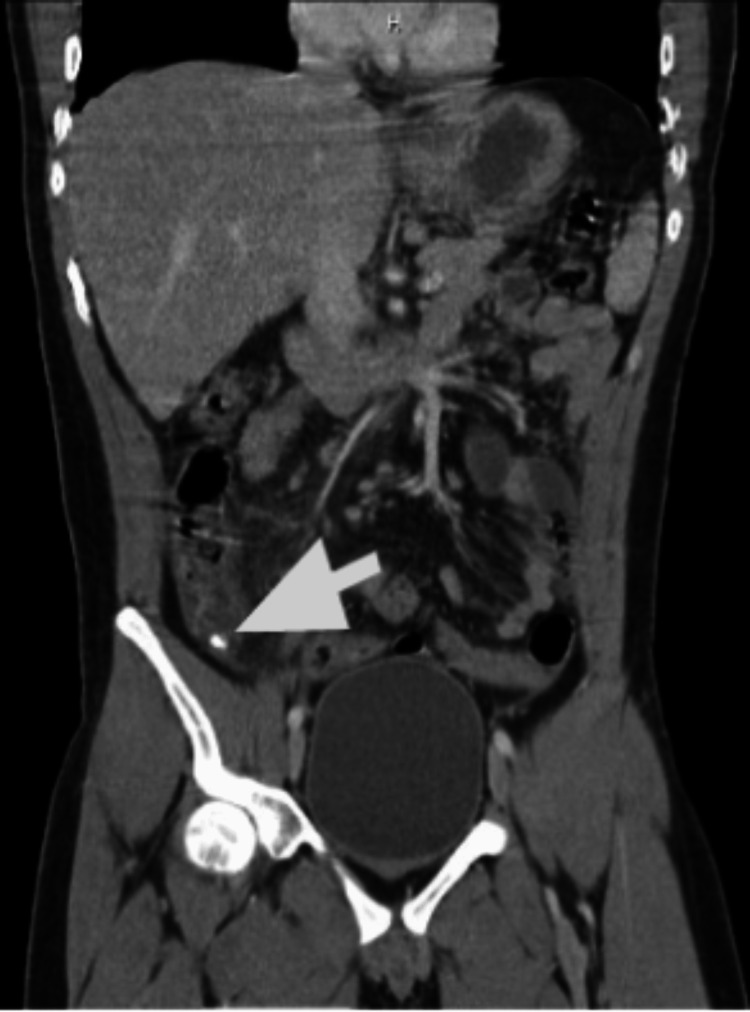
Contrast enhanced CT of the abdomen and pelvis showing acute appendicitis without evidence of perforation

A 22-year-old Latino male presented to the emergency department (ED) with pain in the right lower quadrant. Initial vital signs on presentation were: temperature (T) of 37.6°C, heart rate (HR) of 145, respiratory rate (RR) of 18, blood pressure (BP) of 101/61, and SpO2 95% on room air. Lab studies were significant for leukocytosis (18,000/uL, reference range 4500-11,000/mm), lactic acidosis (2.5 mmol/L, reference range 0.5-2.2 mmol/L), and thrombocytopenia (139 thousand/uL, reference range 150,000-400,000/mm). An abdominal CT scan revealed a dilated appendix with an appendicolith and adjacent fat stranding with no evidence of vascular abnormalities (Figures 1-2). Criteria for systemic inflammatory response syndrome was met prompting subsequent blood cultures that came back positive for Escherichia coli and Proteus mirabilis. Antibiotic sensitivity testing (AST) of Escherichia coli revealed sensitivity to cephalosporins, levofloxacin, ciprofloxacin, amoxicillin/clavulanic acid, and piperacillin/tazobactam with resistance to ampicillin, gentamicin and trimethoprim/sulfamethoxazole. AST of Proteus mirabilis revealed sensitivity to cephalosporins, levofloxacin, ciprofloxacin, gentamicin, and trimethoprim/sulfamethoxazole with no evidence of resistance. Given the diagnostic criteria, the patient was admitted for acute appendicitis. He was given IV piperacillin/tazobactam 3.375 gm and cefoxitin 2000 mg and was taken into the operating room, during which the patient was found to have early gangrenous appendicitis. Laparoscopic appendectomy was uneventful, and the patient was discharged home on postoperative day 3 without repeat blood culture but with a 10-day course of oral cefdinir. 

**Figure 3:**
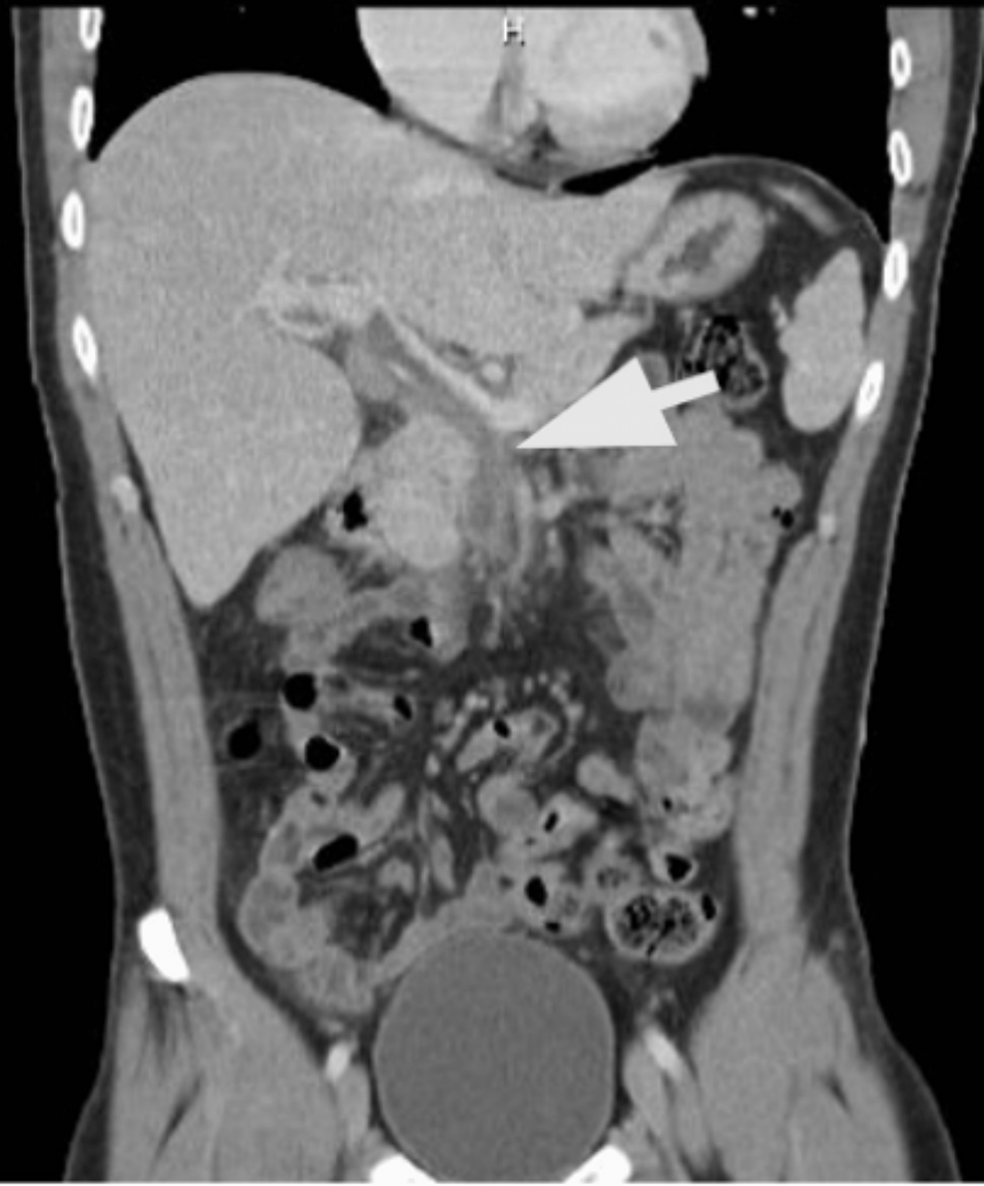
Contrast enhanced CT of the abdomen and pelvis showing venous thrombosis within the superior mesenteric vein and portal vein

**Figure 4:**
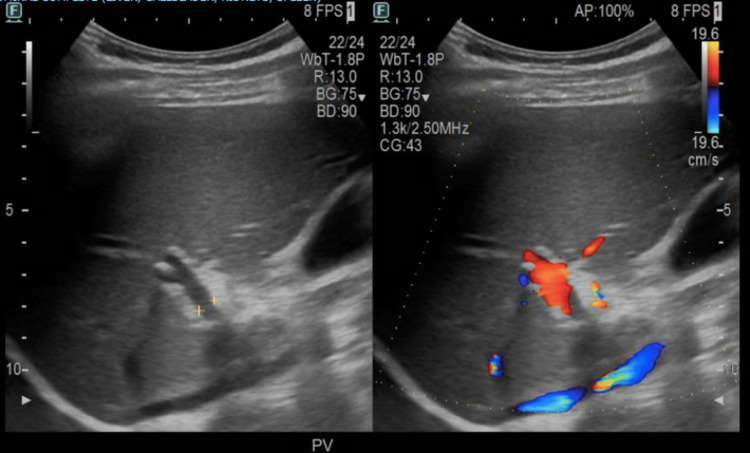
Ultrasound of the abdomen showing resolved venous thrombus in the portal vein

Nine days after discharge, the patient returned to the ED complaining of palpitations. Vital signs on presentation were: T 36.9°C, HR 165, RR 25, BP 96/58, SpO2 96% on room air. Labs revealed leukocytosis (13.5/uL), elevated procalcitonin (3.56, reference range <0.1 ng/ml), elevated total protein (8.7 g/dL, reference range 6.0-7.8 g/dL), elevated liver transaminases (alanine aminotransferase 110 units/L, aspartate aminotransferase 51 units/L), and elevated alkaline phosphatase (ALP) (201 units/L, reference range 25-100 units/L). The collective clinical presentation of elevated temperature, tachycardia, tachypnea, and leukocytosis were positive indicators of sepsis. Prompt IV piperacillin/tazobactam 3.375 gm, heparin 5200 units, and one dose of vancomycin 1000 mg were started while awaiting blood culture results. CT of the abdomen and pelvis demonstrated extensive venous thrombosis involving the main portal vein, superior mesenteric veins, and multiple superior mesenteric vein branches (Figure 3). Bilateral upper and lower extremity duplex scans were negative for deep vein thrombosis. Patient denied any personal and/or family history of coagulopathies. Blood cultures were positive for Escherichia coli and Bacteroides fragilis for which IV levofloxacin 750 mg was administered for targeted therapy while piperacillin/tazobactam was discontinued. A repeat blood culture was obtained on admission day 3 which did not exhibit any growth. After seven days of admission, the patient was discharged with four weeks of metronidazole, levofloxacin, and warfarin. Follow-up US abdomen imaging showed complete resolution of the thrombus (Figure 4).

## Discussion

The most common cause of pylephlebitis is diverticulitis; followed by appendicitis, inflammatory bowel disease, and other intra-abdominal infections. Pylephlebitis is a result of an uncontrolled abdominal infection in regions adjacent to or draining into the portal system [3]. Initially, the small tributaries of the portal system are thrombosed, which then spreads to the larger veins ultimately leading to septic thrombophlebitis of the portal vein [4]. Further complications can then arise such as mesenteric ischemia, infarction, and bowel necrosis. Hypercoagulable states and/or deficiencies in clotting factors are considered major risk factors for pylephlebitis, especially in the setting of sepsis, along with prior abdominal operations, smoking, malignancy, cirrhosis, and the use of steroids [3]. 

Diagnosis and treatment are often delayed due to the majority of patients presenting with nonspecific symptoms including abdominal pain, fever, leukocytosis, and other signs of sepsis or acute abdomen. Currently, there are no diagnostic criteria for pylephlebitis, however, data has shown that advanced imaging has aided in early diagnosis and decreased mortality rates. Both Doppler US and CT can show a thrombus in portal veins. However, CT scans are preferred due to their ability to show additional intra-abdominal processes commonly associated with pylephlebitis, including appendicitis, bowel ischemia, or liver abscesses [5]. 

Bacteremia can also be seen in up to 88% of cases of pylephlebitis; Bacteroides fragilis being the most common microbe along with Escherichia coli and Streptococcus viridans [2]. The lipopolysaccharide and lipid A in Bacteroides expedite blood coagulation, which may play a role in the hypercoagulable state these patients present with [1]. Polymicrobial infections are frequently seen, as in our patient; therefore, blood cultures should always be obtained. 

The main treatment is focused around eradicating all contributing toxic microbes, removing the septic emboli, and treating the source of infection [6]. Currently, there is no set regimen, but empiric antibiotic monotherapy or variations of dual therapy for four weeks have been proven to be successful [7,8]. Another commonly used regimen is two weeks of parenteral antibiotics followed by four weeks of oral antibiotics [2]. Positive blood cultures sourced from intra-abdominal infection warrant source control along with seven days of oral antibiotics targeting specific microbes to prevent potential future complications [9]. The addition of anticoagulative therapy has been a topic of controversy. Data shows it is beneficial to start a patient on anticoagulants if the thrombus has progressed to include the mesenteric veins, if ischemia is noted, or if there is a positive hypercoagulability workup [10]. However, due to its rarity, there is not sufficient data to support the pertinence of anticoagulant therapy and its use should be considered on a case-by-case basis.

Mortality in these patients has decreased tremendously due to the advancements in antibiotic therapy and earlier detection through enhanced imaging. Previous mortality rates were as high as 80%, but in the last decade, rates have been as low as 25% [11].

Currently, there are only three reported cases of pylephlebitis presenting after laparoscopic appendectomy, as evident in our patient [12,13,14]. Though most reported cases of pylephlebitis collected over the past decade have been due to complications of acute appendicitis, it is difficult to determine the actual cause of pylephlebitis in this case. The source of infection was controlled in a timely manner and was treated appropriately with antibiotics tailored to the blood culture sensitivity report. Our patient showed clinical improvement with stable vital signs and improvement of leukocytosis, and therefore, repeat blood culture during his initial admission was not indicated. Patients receiving appropriate antibiotic treatment for bacteremia do not require a repeat blood culture [15]. Repeating cultures, after a positive initial result, was found to be appropriate in certain circumstances: Staphylococcus aureus bacteremia, endovascular infection, candidemia and/or persistent or new infection. However, persistent bacteremia would be clinically present with signs and symptoms, thus a repeat blood culture to confirm sterility of initial bacteremia is not always necessary [16]. Though complete resolution of bacterial sepsis cannot be ruled out, we also cannot rule out the risk laparoscopic procedures have when it comes to thrombotic events. Thus, it is probable that the incomplete resolution of bacterial sepsis predisposed the patient to pylephlebitis but it is also probable that the procedure itself was also a predisposing factor.

## Conclusions

Pylephlebitis is an uncommon yet potentially fatal complication, usually seen in concurrence with ruptured appendicitis. However, it is rarely seen as a complication following acute appendicitis, especially in patients who were treated appropriately with source control and with antibiotics. The nonspecific presentation of this condition makes it difficult to suspect. But once diagnosed, all patients should undergo prompt antibiotic and anticoagulation therapy. Moreover, emphasizing high-resolution CT scans as the standard image modality in patients with high suspicion of intra-abdominal infection may aid in decreasing the likelihood of a missed or delayed diagnosis.
